# Advanced Approach to Information Security Management System Model for Industrial Control System

**DOI:** 10.1155/2014/348305

**Published:** 2014-07-21

**Authors:** Sanghyun Park, Kyungho Lee

**Affiliations:** Center for Information Security Technologies (CIST), Korea University, Seoul 136-713, Republic of Korea

## Abstract

Organizations make use of important information in day-to-day business. Protecting sensitive information is imperative and must be managed. Companies in many parts of the world protect sensitive information using the international standard known as the information security management system (ISMS). ISO 27000 series is the international standard ISMS used to protect confidentiality, integrity, and availability of sensitive information. While an ISMS based on ISO 27000 series has no particular flaws for general information systems, it is unfit to manage sensitive information for industrial control systems (ICSs) because the first priority of industrial control is safety of the system. Therefore, a new information security management system based on confidentiality, integrity, and availability as well as safety is required for ICSs. This new ISMS must be mutually exclusive of an ICS. This paper provides a new paradigm of ISMS for ICSs, which will be shown to be more suitable than the existing ISMS.

## 1. Introduction

In general information systems, almost all security groups use the international information security management system (ISMS) standard which is ISO 27000 series. ISO 27000 series focuses on protection of confidentiality, integrity, and availability of information [1–3]. This ISMS is appropriate for general information systems, where the main threats are dynamic and variable, like malicious hacking.

However, industrial control systems (ICSs) are different from general information systems. While protection from dynamic, variable threats is important on an ICS, safety is most crucial in industrial control [4–6].

**Figure 1:**
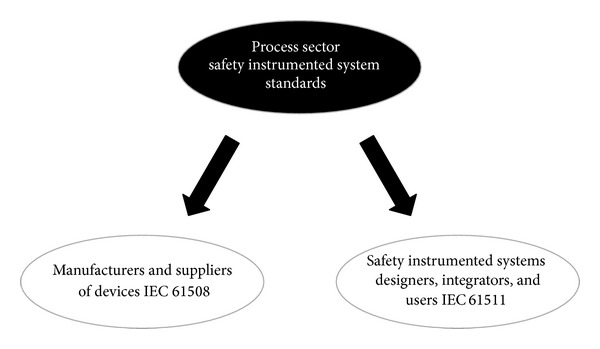
Relationship between IEC 61508 and IEC 61511.

When national infrastructures, like nuclear power plants, deploy an ICS, the ICS is evaluated on the basis of safety [7]. In the field, safety is evaluated by IEC 61508 and IEC 61511. IEC 61508 is the international standard for Functional Safety of Electrical-Electronic-Programmable Electronic Safety-Related Systems and IEC 61511 is the technical standard that defines practices in the engineering of systems that ensures safety of an industrial process (see Figure 1).

ISMS is based on confidentiality, integrity, and availability, and the security needs of ICS are not mutually exclusive because the nature of such businesses is different from general information systems. ICS is of significance in the control of national infrastructures. These systems have unquestionable value, and they must be safe [7, 8]. For this reason, ICSs require safety first, rather than other ISMS based attributes. In the field, process owners for ICSs in fact follow the safety standards IEC 61508 and IEC 61511.

**Figure 2:**
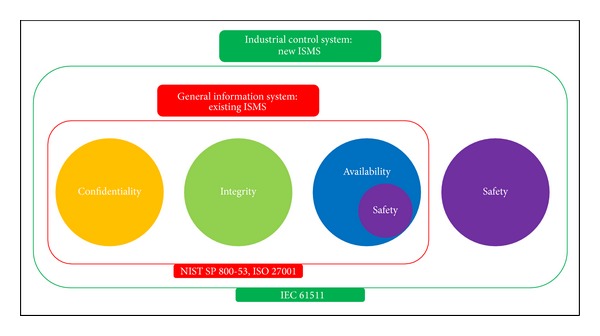
The goal of new information security management system for industrial control system.

In short, it should be configured to a new ISMS based on views of confidentiality, integrity, and availability, as well as safety (see Figure 2).

The ISMS is framework which has presented three views which are confidentiality, integrity, and availability to protect information [1]. However, this paper casts doubt on sufficiency for the three views of existing ISMS to protect assets from internal and external threats and vulnerabilities in ICS.

In case of ICS, social impact due to threats and vulnerabilities like hacking, natural disaster, and internal problems for system cannot compare with general information systems and has great damage that brings out severe economic and social dislocation [4, 5, 8]. Thus, safety becomes the main keyword in ICS.

The requirements of IEC 61511 are based on safety, whereas the requirements and controls of ISO 27001 and NIST SP 800-53 are based on confidentiality, integrity, and availability [7]. When it comes to the safety in ISO 27001 and NIST SP 800-53, it is just a part of availability, so the safety of IEC 61511 is different from the safety of NIST SP 800-53 and ISO 27001.

As a result, this paper suggests that safety presented IEC 61511 should be considered as a part of new ISMS with confidentiality, integrity, and availability. The reason is that information in ICSs could be exposed, leaked, or tweaked if internal safety for system is not guaranteed for unexpected environmental changes like fluctuation of temperature and humidity in ICSs and absence of safety from external threats and vulnerabilities like hacking and natural disaster have a great ripple effect socioeconomically [8, 9].

Therefore, safety should be acknowledged as essential value in ISMS of equal level with confidentiality, integrity, and availability in ICS.

In order to prove this point, we will compare and analyze security controls or requirements of three international standards, namely, ISO 27001, NIST SP 800-53, and IEC 61511. If the safety requirements of IEC 61511, which is followed by people in the ICS field, barely match the security controls that include 21 requirements of ISO 27001, or the security controls of NIST SP 800-53, the ISMS for ICSs, in its present form, is faulty and ineffective [1, 10, 11].

This paper will also compare and analyze common security controls of NIST SP 800-53 that were successfully carried out by the South Korea energy group (thermal, gas, nuclear, combined cycle, electricity, and power exchange) using safety requirements of IEC 61511. The reason for using common security controls to compare with requirements of IEC 61511 is that common security controls are sufficient for every ICS, regardless of the specific application. For these reasons, comparing common security controls and safety requirements of IEC 61511 is essential to further generalize this for every ICS. If the result of matching is the same with the above result of comparison for safety requirements of IEC 61511, security controls of ISO 27001, and security controls of NIST Special Publication 800-53, this analysis can also prove that the ISMS is presently faulty and ineffective in a general ICS environment. In other words, the ISMS that focuses on confidentiality, integrity, and availability of information based on ISO 27000 series is unfit to manage sensitive information on an ICS.

## 2. Introduction of Control Sets for ISO 27001, NIST SP 800-53, and IEC 61511

### 2.1. Domains for Security Controls and Requirements of ISO 27001

**Figure 3:**
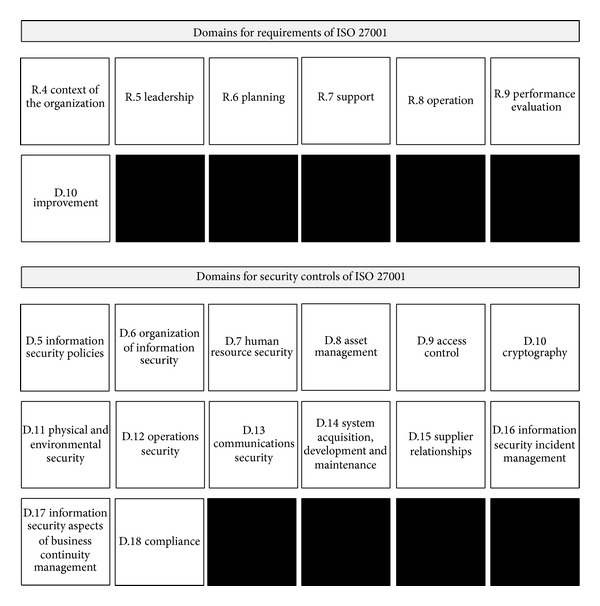
Domains for security controls and requirements of ISO 27001.

ISO 27001 is a document published by ISO and IEC on information technology-security techniques-information security management system-requirements. This document specifies the requirements and security controls for establishing, implementing, maintaining, and continually improving an ISMS within the context of the organization. The security controls presented by ISO 27001 are composed of 34 subdomains in 14 domains. The total number of security controls, which includes 21 requirements, is 140 pieces. The domains for security controls and requirements of ISO 27001 are presented in Figure 3 [1].

### 2.2. Domains for Security Controls of NIST SP 800-53

**Figure 4:**
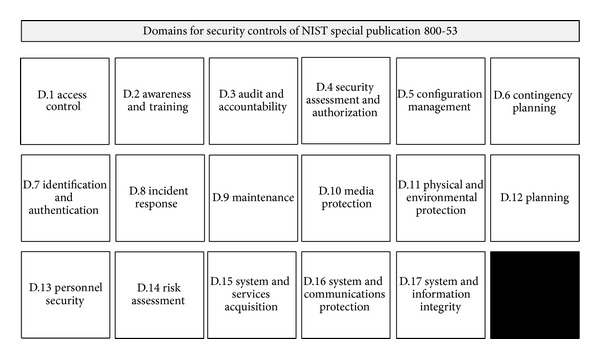
Domains for security controls of NIST SP 800-53.

The NIST Special Publication 800-53 is a document published by NIST for Recommended Security Controls in Federal Information Systems and Organizations. This document especially recommends security controls for ICSs. The recommended security controls are composed of 90 subdomains in 17 domains. The total number of controls is 186 pieces. The domains for recommended security controls are shown in Figure 4 [10].

### 2.3. Domains for Safety Requirements of IEC 61511

**Figure 5:**
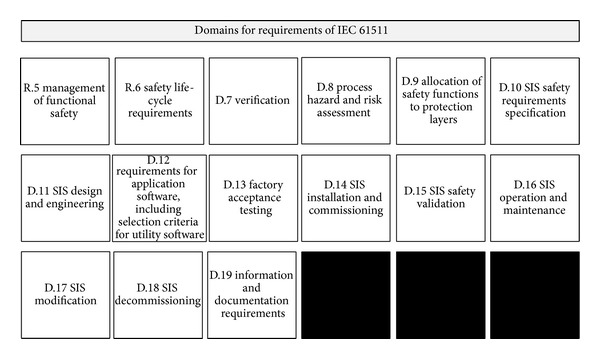
Domains for safety requirements of IEC 61511.

**Figure 6:**
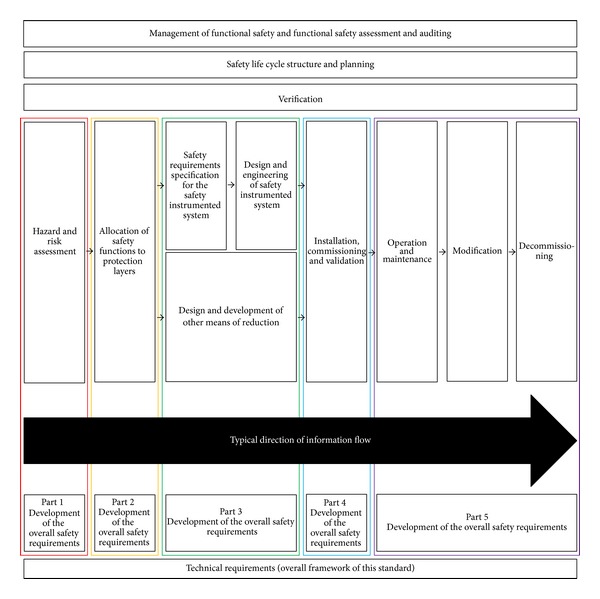
Overall framework of IEC 61511.

IEC 61511 is a technical standard used in the engineering of systems, and it ensures the safety of an industrial process. IEC 61511 consists of 3 chapters. The first chapter is called “framework, definitions, system, hardware and software requirements”; the second chapter is called “guidelines for the application of IEC 61511-1”; and the third chapter is called “guidance for the determination of the required safety integrity levels.” The safety requirements of IEC 61511 are divided into five safety parts and the safety parts consist of development, allocation, design, installation, commissioning, validation, operation, modification, and decommissioning for an ICS. The safety requirements of IEC 61511 are composed of 15 domains and the total number of controls is 215 pieces. The domain for requirements and overall framework of IEC 61511 are shown in Figures 5 and 6 [7].

## 3. Matching Analysis for Security Controls and Requirements of International Standards

Each part of IEC 61511 has several requirements that include the security controls of NIST SP 800-53 or the security controls of ISO 27001.

In order to prove this point, we compare and analyze the security controls/requirements of three international standards, namely ISO 27001, NIST SP 800-53, and IEC 61511, below.

### 3.1. Preparation of Matching Analysis for Security Controls and Requirements of International Standards

We present a comparative security controls list for IEC 61511, ISO 27001, and NIST SP 800-53. The example for list up is presented in Table 1 [1, 7].

### 3.2. Result of Matching Analysis for Security Controls and Requirements of International Standards

In order to find out whether security controls for international standards match, we compare the requirements of IEC 61511 with security controls of NIST SP 800-53 and security controls of ISO 27001.

There are two results based on this comparison. Firstly, the percentage of matching security controls of ISO 27001 with safety requirements of IEC 61511 is 15%. Specifically, the total number of security controls for ISO 27001 is 140 pieces and 21 pieces of these matched with safety requirements of IEC 61511.

Secondly, the percentage of matching security controls for NIST SP 800-53 with safety requirements of IEC 61511 is 16.49%. Specifically, the total number of security controls of NIST SP 800-53 is 194 pieces and 34 pieces of these matched with safety requirements of IEC 61511.

**Figure 7:**
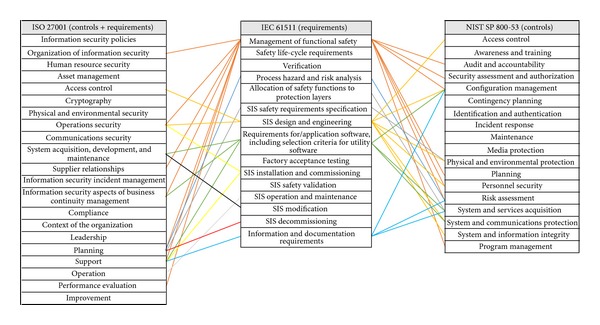
The matching for domains of international standards.

In short, the percentage of matching requirements of IEC 61511, with both security controls of NIST SP 800-53 and security controls of ISO 27001, is quite low. These results mean that ISMS based on ISO 27001 or NIST SP 800-53 is insufficient for a real industrial control system's environment because the ISMS does not reflect specificity for the nature of ICS. The specificity is safety, which is a core value on the IEC 61511 (see Table 2 and Figure 7).

### 3.3. Extracting Items from IEC 61511 to Append New ISMS

The extracting items from IEC 61511 to append new ISMS are selected by certain conditions as follows. The first step is to choose nonmatching requirements of all for IEC 61511 with requirements and controls for NIST SP 800-53 and ISO 27001. The next step is to choose general requirements in each ICS life-cycle types of the nonmatching requirements for IEC 61511 with requirements and controls of NIST SP 800-53 and ISO 27001 and the general requirements are the extracting items. The reason to select general requirements of nonmatching requirements is to maintain a level of requirements and controls with ISO 27001 and NIST SP 800-53 and assure safety for new ISMS [12, 13].

The recommended extracting items of safety from IEC 61511 to develop new ISMS are shown in Table 3.

This paper presents that the safety has two meanings broadly. The first meaning is safety against external factors like hacking and natural disaster; another is safety against internal factors like internal failure for system.

The requirements of IEC 61511 and the requirements of ISO 27001 and NIST SP 800-53 do not present direct requirements against internal and external threats and vulnerabilities to hinder safety in ICS. Instead, requirements of IEC 61511 present safety requirements in each ICS life-cycle types that guarantee safety from the internal and external threats and vulnerabilities, and the safety requirements aim to improve safety for ICS that is core to manage well risk from the internal and external threats and vulnerabilities.

## 4. Matching Analysis for Common Security Controls of NIST SP 800-53 in South Korea Energy Industry and Safety Requirements of IEC 61511

Each part of IEC 61511 has several requirements that include the security controls of NIST SP 800-53. In this section, we will not compare and analyze whole security controls of international standards, but instead we will compare and analyze common security controls of NIST SP 800-53 that were successfully carried out by the South Korea energy group (thermal, gas, nuclear, combined cycle, electricity, and power exchange) with safety requirements of IEC 61511. This is because entire security controls of NIST SP 800-53 do not apply to the South Korea energy group.

In order to find out the common security controls from the entire security controls of NIST SP 800-53, we constructed evaluation frame that has security controls of NIST SP 800-53. We asked the South Korea energy group, that is, power exchange, electricity, gas, combined cycle, nuclear, and thermal groups, to fill out a questionnaire [10, 11, 14] (see Table 4).

### 4.1. The Data Gathering to Find Out Common Security Controls of NIST SP 800-53 in South Korea Energy Industry

In order to gather data, we drew up an evaluation sheet for the security controls based on the NIST Special Publication 800-53 that includes security guidance and recommends security controls for ICSs [15–18].

**Figure 8:**
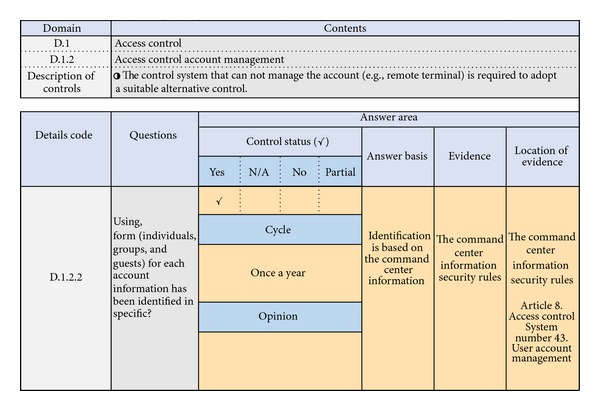
Example of an evaluation sheet.

The evaluation sheet is shown in Figure 8.

Answers for each item are classified as yes, no, partial, and N/A. Developers, operators of energy management system, and process owners filled up the questionnaire.

### 4.2. The Result for Common Security Controls of NIST SP 800-53 in South Korea Energy Industry

We compared and analyzed the current security controls status for the South Korea energy group (thermal, gas, nuclear, combined cycle, electricity, and power exchange) and then collected a common security controls mean, that is, controls for every South Korea group to carry out successfully. The common security controls are as show in Table 5.

### 4.3. Results of Matching Analysis for Common Security Controls of NIST SP 800-53 in South Korea Energy Groups and Requirements IEC 61511

The safety requirements of IEC 61511 match common security controls of NIST SP 800-53. In fact, it may be more difficult to match the safety requirements of IEC 61511 with common security controls of NIST SP 800-53 due to the nature of the standard. The standard generalizes requirements, while the value for common security controls of NIST SP 800-53 compare well enough with the safety requirements of IEC 61511 (see Table 6).

It is difficult to match common security controls of NIST SP 800-53 with safety requirements of IEC 61511 perfectly; however, the safety requirements of IEC 61511 match with common security controls. In other words, it is not hard to include safety as an ICS attribute.

The point of this paper is that the safety emphasized on IEC 61511 can reflect information security management system for ICS.

## 5. Conclusions

This paper presented two methodologies to prove that a new information security management system based on confidentiality, integrity, availability, and safety is required on the industrial control system.

**Figure 9:**
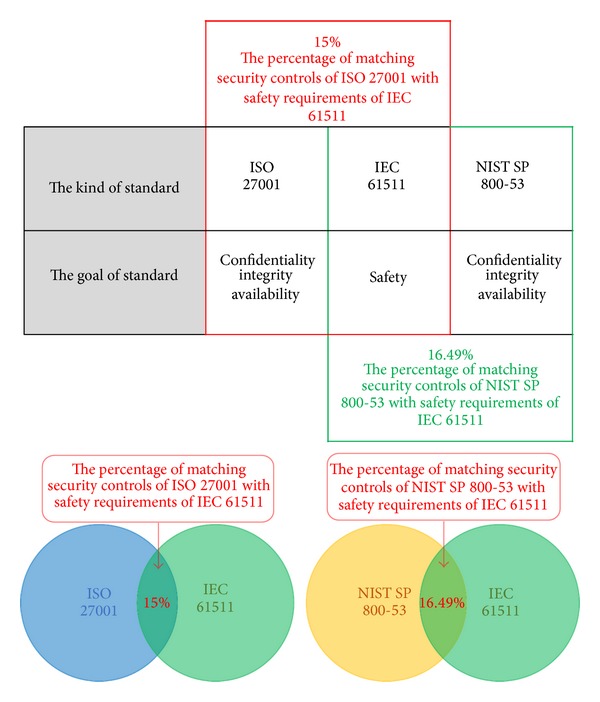
Comparative analysis for controls of international standards.

The first methodology was analysis of matching security controls with international standards. From the first methodology, it was seen that the percentage of matching between the requirements of IEC 61511, the security controls of NIST SP 800-53, and the security controls of ISO 27001 is very low. These results mean that ISMS based on ISO 27001 or NIST SP 800-53 is insufficient to make for real ICSs because the ISMS does not reflect specificity of the nature of ICSs (see Figure 9).

The second methodology involved analysis of matching of the common security controls of NIST SP 800-53 that were successfully carried out by the South Korea energy group (thermal, gas, nuclear, combined cycle, electricity, and power exchange) with the safety requirements of IEC 61511. These results showed that it is difficult to match common security controls of NIST SP 800-53 in South Korea with safety requirements of IEC 61511 perfectly. However, the safety requirements of IEC 61511 match reasonably well with common security controls. In other words, it is not hard for safety to be included in an industrial control system.

The ICS is different from a general information system and an ISMS based on confidentiality, integrity, and availability never achieves mutually exclusive security policy for an ICS.

Just as integrity is significant for finance and confidentiality is significant for manufacturing, safety is significant for ICSs [3, 5, 6]. This paper proves that safety is very significant for ICSs, and safety should be included in an ISMS based on confidentiality, integrity, and availability of information.

In brief, a new ISMS based on confidentiality, integrity, and availability as well as safety is required in ICSs. This new information security management system is mutually exclusive to the nature of industrial control system.

We expect that the performance of information security for ICSs will be improved through our work.

## Figures and Tables

**Table 1:** The example of security controls list up for international standard.

Domain number	IEC 61511	Domain number	ISO 27001
5.2.2.1	Persons, departments, organizations, or other units which are responsible for carrying out and reviewing each of the safety life-cycle phases shall be identified and be informed of the responsibilities assigned to them (including, where relevant, licensing authorities or safety regulatory bodies).	A.6.1.1	All information security responsibilities shall be defined and allocated.

**Table 2:** The matching analysis for requirements or security controls of international standards.

	Comparison targets	
	ISO 27001	NIST SP 800-53
The total number of security controls and requirements	140 (requirements: 21, security controls: 114)	194
The total number of matching security controls for comparison target with safety requirements of IEC 61511	21	32
The percentage of matching security controls for comparison target with safety requirements of IEC 61511	15%	16.49%

**Table 3:** The example of recommended extracting items from IEC 61511.

	Recommended extracting items
IEC 61511	The safety requirements shall be derived from the allocation of safety instrumented functions and from those requirements identified during safety planning.
	The need for a factory acceptance testing should be specified during the design phase of a project.
	Installation and commissioning planning shall define all activities required for installation and commissioning. The planning shall provide the following: (i) the installation and commissioning activities; (ii) the procedures, measures, and techniques to be used for installation and commissioning; (iii) when these activities shall take place; (iv) the persons, departments, and organizations responsible for these activities. Installation and commissioning planning may be integrated in the overall project planning where appropriate.
	The validation of the safety instrumented system and its associated safety instrumented functions shall be carried out in accordance with the safety instrumented system validation planning.
	⋮
	Discrepancies between expected behaviour and actual behaviour of the SIS shall be analysed and, where necessary, modifications made such that the required safety is maintained. This shall include monitoring the following: (i) the actions taken following a demand on the system; (ii) the failures of equipment forming part of the SIS established during routine testing or actual demand; (iii) the cause of the demands; (iv) the cause of false trips.
	The procedures shall include a clear method of identifying and requesting the work to be done and the hazards which may be affected (modification and decommissioning).
	Modification shall be performed with qualified personnel who have been properly trained. All affected and appropriate personnel should be notified of the change and trained with regard to the change.

**Table 4:** The domain and subdomain of NIST SP 800-53 for an ICS.

Domain	Subdomain
D.1 Access Control	D.1.2 Account Management
	D.1.3 Access Enforcement
	D.1.5 Separation of Duties
	D.1.6 Least Privilege
	D.1.7 Unsuccessful Login Attempts
	D.1.8 System Use Notification
	D.1.10 Concurrent Session Control
	D.1.11 Session Lock
	D.1.17 Remote Access
	D.1.18 Wireless Access
	D.1.19 Access Control for Mobile Devices
	D.1.22 Publicly Accessible Content

D.2 Awareness and Training	D.2.2 Security Awareness
	D.2.3 Security Training

D.3 Audit and Accountability	D.3.2 Auditable Events
	D.3.3 Response to Audit Processing Failures
	D.3.4 Audit Reduction and Report Generation
	D.3.5 Audit Generation

D.4 Security Assessment and Authorization	D.4.2 Security Assessments
	D.4.7 Continuous Monitoring

D.5 Configuration Management	D.5.3 Configuration Change Control
	D.5.4 Security Impact Analysis
	D.5.5 Access Restrictions for Change
	D.5.6 Configuration setting
	D.5.7 Least Functionality

D.6 Contingency Planning	D.6.2 Contingency Plan
	D.6.4 Contingency Plan Testing and Exercises
	D.6.10 Information System Recovery and Reconstitution

D.7 Identification and Authentication	D.7.2 Identification and Authentication (Organizational Users)
	D.7.3 Device Identification and Authentication
	D.7.4 Identifier Management
	D.7.5 Authenticator Management
	D.7.7 Cryptographic Module Authentication

D.8 Incident Response	D.8.6 Incident Reporting

D.9 Maintenance	D.9.4 Non-Local Maintenance

D.10 Media Protection	D.10.5 Media Transport

D.11 Physical and Environmental Protection	D.11.3 Physical Access Control

D.12 Planning	D.12.2 System Security Plan

D.14 Risk Assessment	D.14.2 System Categorization
	D.14.3 Risk Assessment
	D.14.5 Vulnerability Scanning

D.15 System and Services Acquisition	D.15.4 Acquisitions
	D.15.8 Security Engineering Principles

D.16 System and Communications Protection	D.16.2 Application Partitioning
	D.16.3 Security Function Isolation
	D.16.7 Boundary Protection
	D.16.8 Transmission Integrity
	D.16.9 Transmission Confidentiality
	D.16.10 Network Disconnect
	D.16.12 Cryptographic Key Establishment and Management
	D.16.13 Use of Cryptography
	D.16.14 Public Access Protections
	D.16.15 Collaborative Computing Devices
	D.16.19 Voice Over Internet Protocol
	D.16.20 Secure Name/Address Resolution Service (Authoritative Source)
	D.16.21 Secure Name/Address Resolution Service (Recursive or Caching Resolver)
	D.16.22 Architecture and Provisioning for Name/Address Resolution Service
	D.16.23 Session Authenticity

D.17 System and Information Integrity	D.17.2 Flaw Remediation
	D.17.3 Malicious Code Protection
	D.17.4 Information System Monitoring
	D.17.6 Security Functionality Verification
	D.17.7 Software and Information Integrity
	D.17.8 Spam Protection

**Table 5:** The List of Common Security Controls in South Korea Energy Industry for NIST SP 800-53.

Number	Main domain name	Subdomain name	Code of security control	Security control
1	Access control	Account management	AC-2	The organization manages information system accounts, including identifying account types.
2		Separation of duties	AC-5	The organization implements separation of duties through assigned information system access authorizations.
3		Least privilege	AC-6	The organization employs the concept of least privilege, allowing only authorized accesses for users which are necessary to accomplish assigned tasks in accordance with organizational missions and business functions.

4	Media protection	Media access	MP-2	The organization restricts access to [Assignment: organization-defined types of digital and non-digital media] to [Assignment: organization-defined list of authorized individuals] using [Assignment: organization-defined security measures].
5		Media marking	MP-3.a	The organization marks, in accordance with organizational policies and procedures, removable information system media and information system output indicating the distribution limitations, handling caveats, and applicable security markings (if any) of the information.
6			MP-3.b	The organization exempts [Assignment: organization-defined list of removable media types] from marking as long as the exempted items remain within [Assignment: organization-defined controlled areas].
7		Media storage	MP-4.a	The organization physically controls and securely stores [Assignment: organization-defined types of digital and non-digital media] within [Assignment: organization-defined controlled areas] using [Assignment: organization-defined security measures].
8			MP-4.b	The organization protects information system media until the media are destroyed or sanitized using approved equipment, techniques, and procedures.
9		Media transport	MP-5.a	The organization protects and controls [Assignment: organization-defined types of digital and non-digital media] during transport outside of controlled areas using [Assignment: organization-defined security measures].
10			MP-5.c	The organization restricts the activities associated with transport of such media to authorized personnel.

11	Physical and environmental protection	Physical access authorizations	PE-2	The organization develops and keeps a current list of personnel with authorized access to the facility where the information system resides (except for those areas within the facility officially designated as publicly accessible).
12		Monitoring physical access	PE-6.a	The organization monitors physical access to the information system to identify and respond to physical security incidents.
13			PE-6.b	The organization reviews physical access logs [Assignment: organization-defined frequency].
14		Visitor control	PE-7	The organization controls physical access to the information system by authenticating visitors before authorizing access to the facility where the information system resides, other than areas designated as publicly accessible.
15		Emergency shutoff	PE-10	The organization provides the capability of shutting off power to the information system, or individual system components, in emergency situations.
16		Emergency lighting	PE-12	The organization employs and maintains automatic emergency lighting for the information system that activates in the event of a power outage or disruption and that covers emergency exits and evacuation routes within the facility.
17		Fire protection	PE-13	The organization employs and maintains fire suppression and detection devices/systems for the information system that are supported by an independent energy source.
18		Temperature and humidity controls	PE-14	The organization maintains temperature and humidity levels within the facility where the information system resides at [Assignment: organization-defined acceptable levels].
19		Water damage protection	PE-15	The organization protects the information system from damage resulting from water leakage by providing master shutoff valves that are accessible, working properly, and known to key personnel.
20		Location of information system Components	PE-18	The organization positions information system components within the facility to minimize potential damage from physical and environmental hazards and to minimize the opportunity for unauthorized access.

21	System and communications protection	Denial of service protection	SC-5	The information system protects against or limits the effects of the following types of denial of service attacks: [Assignment: organization-defined list of types of denial of service attacks or reference to source for current list].
22		Boundary protection	SC-7.a	The information system monitors and controls communications at the external boundary of the system and at key internal boundaries within the system.
23			SC-7.b	The information system connects to external networks or information systems only through managed interfaces consisting of boundary protection devices arranged in accordance with an organizational security architecture.

**Table 6:** Comparison for common security controls of NIST SP 800-53 and safety requirements of IEC 61511.

Number	Main domain name	Code of security control for common security controls in South Korea Energy Industry	Safety requirements of IEC 61511
1	Access control	AC-2	〈**R**.11.7.2.4〉 Enabling and disabling the read-write access shall be carried out only by a configuration or programming process using the maintenance/engineering interface with appropriate documentation and security measures.
2		AC-5	
3		AC-6	

4	Media protection	MP-2	—
5		MP-3.a	
6		MP-3.b	
7		MP-4.a	
8		MP-4.b	
9		MP-5.a	
10		MP-5.c	

11	Physical and environmental protection	PE-2	〈**D**.11.2.11〉 For subsystems that on loss of power do not fail to the safe state, all of the following requirements shall be met and action taken according to 11.3: (i) loss of circuit integrity is detected (for example, end-of-line monitoring); (ii) power supply integrity is ensured using supplemental power supply (for example, battery back-up and uninterruptible power supplies); (iii) loss of power to the subsystem is detected.
12		PE-6.a	
13		PE-6.b	
14		PE-7	
15		PE-10	
16		PE-12	
17		PE-13	
18		PE-14	
19		PE-15	
20		PE-18	

21	System and communications protection	SC-5	〈**D**.11.7.3.3〉 The communication interface shall be sufficiently robust to withstand electromagnetic interference including power surges without causing a dangerous failure of the SIF.
22		SC-7.a	
23		SC-7.b	
